# Effects of Animal-Based Foods on Metabolic Outcomes in Adults with MASLD and Comorbidities: A Systematic Review of Randomized Controlled Trials (2020–2026)

**DOI:** 10.3390/nu18101508

**Published:** 2026-05-08

**Authors:** Joanna Michalina Jurek, Katarzyna Zablocka-Slowinska, Joanna Pieczynska, Anna Lipert, Helena Clavero-Mestres, Teresa Auguet

**Affiliations:** 1 Grup de Recerca GEMMAIR (AGAUR)-Medicina Aplicada (URV), Departament de Medicina i Cirurgia, Universitat Rovira i Virgili (URV), Institut d’Investigació Sanitària Pere Virgili (IISPV), Mallafré Guasch, 4, 43007 Tarragona, Spain; helena.clavero@urv.cat (H.C.-M.); tauguet.hj23.ics@gencat.cat (T.A.); 2 Department of Biostatistics and Translational Medicine, Medical University of Lodz, Mazowiecka 15, 92-215 Lodz, Poland; 3 The Faculty of Finance and Management, WSB Merito University Wroclaw, Fabryczna 29/31, 53-609 Wroclaw, Poland; 4 Department of Dietetics and Bromatology, Wroclaw Medical University, Borowska 211, 50-556 Wroclaw, Poland; joanna.pieczynska@interia.pl; 5 Cardiovascular and Metabolic Diseases Prevention Laboratory, Department of Preventive Medicine, Medical University of Lodz, 92-213 Lodz, Poland; 6 Servei Medicina Interna, Hospital Universitari de Tarragona Joan XXIII, Mallafré Guasch, 4, 43007 Tarragona, Spain

**Keywords:** animal-based foods, MASLD, T2DM, metabolic syndrome, dairy products, meat, fish, milk, hepatic steatosis, hepatic fibrosis, inflammation, glycemic control, lipid profile, body weight, systematic review

## Abstract

Background: Metabolically dysfunction-associated steatotic liver disease (MASLD) is a growing public health challenge linked to obesity and metabolic dysregulation. Since pharmacological options are limited, Mediterranean (MED) and DASH patterns are recommended. These diets include animal-derived foods, providing essential nutrients but also potential saturated fats. Objective: This updated systematic review aims to evaluate evidence from randomized controlled trials (RCTs) regarding the effects of specific animal-based foods on metabolic, hepatic, and anthropometric outcomes in adults with MASLD. Methods: A systematic search of nine databases (including PubMed, Scopus, and Embase) and trial registries (ClinicalTrials.gov) was conducted for RCTs published between January 2020 and 31 March 2026. Participants were adults (18–65 years) with MASLD. Interventions included animal-derived foods typical of MED/DASH. Outcomes focused on hepatic function, lipid profiles, glycemic control, and anthropometry. Risk of bias was assessed using the Cochrane RoB 2.0 tool. Results: A total of seven RCTs demonstrated food-specific and heterogeneous effects. Freshwater fish and vitamin D-fortified probiotic yogurt showed consistent benefits for glycemic control and lipid profiles. Fish oil and omega-3 supplementation led to modest improvements in lipids and hepatic markers. Ghee intake was linked with improved total cholesterol and liver enzyme (ALP). Red meat demonstrated heterogeneous effects depending on dose and background dietary pattern. Most studies exhibited limited concerns regarding risk of bias. Discussion: Animal-based foods within MED/DASH patterns exert distinct effects in MASLD. Freshwater fish and fortified yogurt appear most beneficial. The evidence is limited by small sample sizes in specific food categories and variability in dietary assessment methods. These findings support nuanced, evidence-based recommendations for MASLD management.

## 1. Introduction

Metabolically dysfunction-associated steatotic liver disease (MASLD), formerly known as non-alcoholic fatty liver disease (NAFLD), is an emerging chronic hepatic condition that is being diagnosed among 30–38% of adults worldwide, with prevalence in Europe reaching 25–30% [1,2]. The recent change in nomenclature aims to better reflect the underlying metabolic pathophysiology, reduce stigmatization, and enhance disease recognition. Under this updated framework, individuals previously classified as having NAFLD now fall within the MASLD definition, which replaces the earlier exclusionary criterion based solely on non-alcohol intake with broader cardiometabolic risk factors. This shift aligns with the rising global burden of metabolic disease, with prevalence estimates increasing from ~25% in the 1990s to nearly 40% in the late 2010s, along with the rise in obesity and type 2 diabetes mellitus (T2DM) [2]. The pathophysiology of MASLD is multifactorial, and beyond hepatic complications, it involves overlapping disturbances in glucose and lipid metabolism. Insulin resistance, dyslipidemia, and visceral adiposity promote triglyceride accumulation and oxidative stress in hepatocytes, while chronic low-grade inflammation contributes to hepatocellular injury and fibrogenesis [3]. Disruption of the gut–liver axis, including microbiota alterations and lipopolysaccharide translocation, further amplifies immune activation and hepatic inflammation, collectively accelerating disease progression from steatosis to steatohepatitis, fibrosis, cirrhosis, and finally hepatic carcinoma [1,3]. Patients with MASLD have an increased risk of cardiovascular and renal disease, with global mortality estimated at 12.6 per 1000 person-years, of which 4.2 per 1000 are cardiovascular-related and 0.9 per 1000 are liver-related [2]. MASLD is projected to become the leading indication for liver transplantation with a significant economic burden, even though pharmacological treatments with resmetirom and semaglutide were recently approved for selected MASH populations with moderate-to-advanced fibrosis [4,5]. Thus, for the majority of patients with MASLD, lifestyle modification remains the first-line therapy.

Clinical guidelines recommend dietary intervention as the cornerstone of management, with structured patterns such as the Mediterranean diet (MedDiet), Nordic diet, and Dietary Approaches to Stop Hypertension (DASH) consistently showing improvements in hepatic steatosis, insulin resistance, and inflammation [6,7,8]; the specific contribution of individual food groups remains an area of growing interest. Although these dietary patterns are predominantly plant-based, selected animal-sourced foods characteristic of the Mediterranean, DASH, and Nordic diets (e.g., fish and seafood, fermented dairy products, and lean meats) also provide important nutrients and may exert independent health-promoting effects.

Animal-based foods represent a heterogeneous dietary category with variable metabolic effects. Red and processed meats, rich in saturated fat, cholesterol, advanced glycation end-products (AGEs), and heme iron, have been linked to higher risks of hepatic steatosis, insulin resistance, and fibrosis, contributing to disease progression [9,10,11]. Contrastingly, lean poultry and white meats show more neutral associations, though some studies suggest sex-specific effects, with protective outcomes in men but not women [10]. In addition, fish and fatty species rich in long-chain omega-3 polyunsaturated fatty acids, eicosapentaenoic acid (EPA), and docosahexaenoic acid (DHA) demonstrate beneficial effects on insulin sensitivity, triglycerides, and systemic inflammation. Results of clinical studies further confirmed reductions in triglycerides, HOMA-IR, waist circumference, and aspartate aminotransferase (AST), although effects on alanine aminotransferase (ALT), gamma-glutamyl transpeptidase (GGTP), and BMI remain inconsistent [12], whereas trials conducted in MASLD with T2DM showed improvements in fatty liver index, visceral adiposity, and lipid accumulation following omega-3 supplementation [11].

Similarly to fish, dairy products demonstrated inconsistent health effects that varied between the types. Low- to medium-fat dairy, especially fermented beverages like yogurt and kefir, being rich in protein, calcium, vitamin D, and probiotics, has been associated with reduced risk of MASLD and cirrhosis [13,14,15]. These effects are consistent with observations from the large-scale cohort studies suggesting that dairy-rich diets lower MASLD incidence, independent of genetic predisposition [13]. Moderate intake of fermented or low-fat dairy has also been linked to improvements in the gut–liver axis function and systemic inflammation [15,16]. The impact of full-fat dairy remains debated, but emerging evidence suggests that the “dairy matrix” may attenuate adverse effects, resulting in neutral or even protective outcomes when consumed in moderation along with a healthy and balanced diet [15].

Taken together, current evidence indicates that different foods of animal origin may exert various metabolic effects, which should be further investigated. Despite the growing body of evidence supporting the use of dietary recommendations based on using model diets in patients with MASLD, the independent metabolic and hepatic effects of specific animal-derived foods remain insufficiently characterized, with available clinical trials showing heterogeneous and often inconsistent results. While red and processed meats are linked to adverse outcomes, fish and fish-derived oils and fermented dairy products appear to be protective. Moreover, recent RCTs evaluating individual animal-based foods and protein preparations in MASLD, particularly in those with coexisting obesity and/or T2DM, have not yet been systematically synthesized under the updated MASLD framework. Therefore, the main objective of this systematic review is to synthesize and evaluate the recent evidence on the effects of individual animal-derived foods, animal-based food groups, and animal protein preparations on metabolic, inflammatory, and hepatic outcomes in adults aged 18–65 years with a confirmed diagnosis of MASLD, with or without obesity, metabolic syndrome, or type 2 diabetes, from identified RCTs published between 1 January 2020 and 31 March 2026. This review builds upon previous systematic reviews covering studies prior to 2020, the results of which are discussed in the remainder of this work. The main purpose of this analysis is to provide updated evidence to guide dietary recommendations, support long-term weight, glycemic, and lipid control, and improve liver health, while avoiding unnecessary restrictions that may reduce patient compliance.

## 2. Materials and Methods

The methodology of this review was based on the Preferred Reporting Items for Systematic Reviews and Meta-Analyses (PRISMA) principles for systematic reviews [17].

The protocol of this systematic review was registered in PROSPERO with the number CRD420251135067 and can be accessed in the PROSPERO 2025 database [Available from https://www.crd.york.ac.uk/PROSPERO/view/CRD420251135067] (13 February 2026). The objectives for this study were defined based on the framework involving patients, intervention, comparison, and outcomes (PICOS) and were defined to investigate how foods of animal origin and/or food groups can impact metabolic outcomes in MASLD alone and with other comorbidities, including obesity, metabolic syndrome, and T2DM (Table 1).

### 2.1. Search Strategy

Search terms were developed a priori, informed by systematic screening of relevant terminology used in published studies, and were further identified by the online Pubmed tool, allowing for screening relevant terms in published studies (Pubmed MeSH). We searched in 9 databases, including Google Scholar, to identify supplementary records and clinical trial registers. The search strategy was based on the use of combinations demonstrated in Table 2.

No additional regulatory databases or online repositories beyond those listed were searched. We did not contact any organizations, manufacturers, or individuals to identify studies. We did not examine reference lists, conduct backward or forward citation searching, or hand-search journals or conference proceedings beyond the database searches described above.

To ensure comprehensive retrieval, we constructed a systematic algorithm that combined disease-related terms with dietary keywords using Boolean operators. All search term combinations were adapted to database-specific syntax, and no date restrictions were imposed to maximize sensitivity.

The searching criteria were agreed upon between three researchers, who took part in searching databases selected to identify Randomized Control Trials (RCTs) published between 1 January 2020 and 31 March 2026. The specific time limits were applied in order to capture the most recent and clinically relevant evidence on dietary interventions in MASLD, reflecting current diagnostic criteria, assessment methods, and nutritional approaches, thus ensuring that the findings of this review are based on the recent trials.

### 2.2. Study Selection

The manuscript screening and selection process involved three reviewers, each of whom independently assessed all identified records (titles and abstracts) to ensure consistent application of the inclusion criteria. The full texts of potentially eligible RCTs were then evaluated independently by the same three reviewers, and any disagreements were resolved through discussion, including joint online review of the manuscripts when needed. After final study selection, data extraction was performed in duplicate by at least two independent reviewers using a predefined extraction template aligned with MASLD diagnostic and metabolic outcome criteria [18], and a third reviewer verified all entries for accuracy and completeness. Any discrepancies in extracted values, variable definitions, or outcome categorization were resolved by cross-checking the original manuscripts and reaching agreement through discussion.

### 2.3. Selection Criteria

We predefined the eligibility criteria a priori to ensure a transparent and reproducible study selection process. The selection criteria were applied as in the methodology already employed in previous work published [19,20]. Briefly, this systematically conducted review was focused solely on the RCTs that investigated dietary interventions with animal-based single foods, food groups, or powdered forms of these products in adults aged between 18 and 65 years. Eligible participants were diagnosed with MASLD using validated methods such as liver biopsy or ultrasonography, either alone or in combination with other metabolic conditions, including obesity, metabolic syndrome, T2DM, or insulin resistance. Studies were excluded if they: (1) evaluated supplements, medications, or plant-based products; (2) investigated model dietary patterns; (3) enrolled participants younger than 18 or older than 65 years or included pregnant women; (4) were published outside the predefined time frame (1 January 2020–31 March 2026); (5) employed case–control or observational study designs; or (6) combined dietary interventions with broader lifestyle modifications (e.g., behavioral or cognitive-behavioral therapies, psychoeducational programs, self-management strategies, motivational interviewing, physical activity, sleep hygiene, stress management, or changes in diet quality and habits).

Studies were considered for inclusion if they met all inclusion criteria and none of the exclusion criteria presented in Table 3.

**Figure 1:**
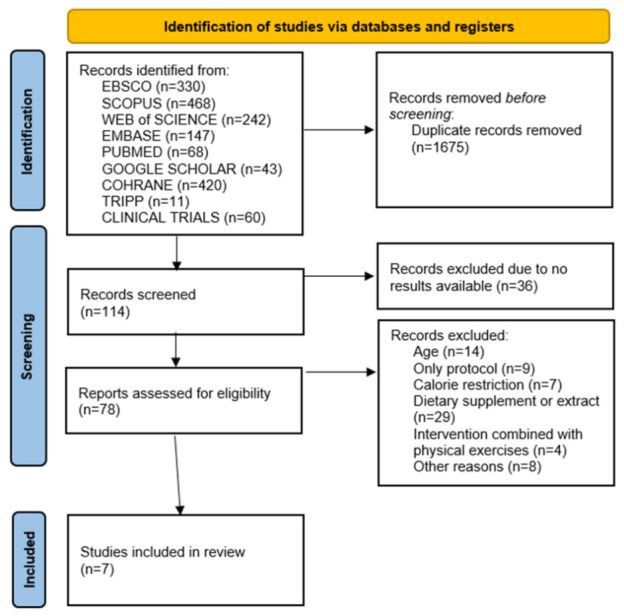
PRISMA chart presenting the overview of systematic searches applied in this review process, along with the number of identified studies at each step.

The systematic search initially identified 1789 manuscripts of potential interest, including 1718 papers retrieved from the searched databases and 71 from clinical trial registries (in detail, 60 from ClinicalTrials.gov, 11 studies in TRIPP, 242 studies from Web of Science, 468 studies from Scopus, 147 studies from EMBASE, 43 studies from Google Scholar, 330 from EBSCO, and 68 from Pubmed). After removing duplicates, 114 records remained for screening. Although we initially identified 78 studies that seemed to meet the screening criteria, such as publication date within the given timeframe and population of interest, the age of participants was the major factor why 14 studies were excluded (the age of patients was 70–75 years). As studies were newly established, most of them had only protocols available with no results published. Some interventions had additional modifiable factors, such as a calorie-restricted diet (7 studies), which were combined with physician activity (4 studies), or the animal-based food was given as a supplement or extract, a common case for fish oils and bee pollen (29 studies). Following that eligibility assessment, finally, 7 relevant RCTs were included in this review. The study selection process was conducted using the PRISMA 2020 online tool [17] and is presented in Figure 1.

### 2.4. Data Extraction

Data extracted from each identified study were reported in an Excel file and included the following variables: DOI, study title, first author’s family name, publication year, study design, and geographical region where the study was conducted. For each dietary intervention, information was recorded on the specific food or food group examined, intervention duration in weeks, and the type of comparator used. Baseline and post-intervention values were collected for all relevant metabolic outcomes, including anthropometric indicators (age, body weight, BMI, waist circumference, body fat percentage, and diastolic and systolic blood pressure), glucose metabolism parameters (fasting glucose, fasting insulin, HOMA-IR, and HbA1c), lipid profile markers (triglycerides, total cholesterol, LDL-C, and HDL-C), and inflammatory biomarkers (hs-CRP and LPS). Liver-related outcomes were also comprehensively captured, encompassing hepatic enzymes (AST, ALT, ALP), quantitative measures of hepatic steatosis (CAP, HIS, FLI, and fatty liver grade), and liver fibrosis assessed by FibroScan.

Prespecified outcomes were extracted at the study-arm level at baseline and at the end of the intervention (post-intervention). When multiple post-baseline time points were reported, the end-of-intervention assessment was selected a priori. Between-group changes were prioritized because they account for changes in the control group and thus isolate the effect attributable to the animal-based intervention. This approach yields more valid and clinically interpretable outcomes of efficacy by reducing bias from disease course and/or placebo effect. Outcome data were recorded using the summary statistics provided in the original reports (mean ± SD or median). When both intention-to-treat (ITT) and per-protocol (PP) analyses were available, intention-to-treat results were preferentially extracted; otherwise, the primary analysis defined by the trial authors was used. Outcomes not reported for a given arm were documented as not available. No minimally important difference or effect-size magnitude categories were prespecified due to heterogeneity in results and absence of meta-analysis. No data conversion to alternative effect measures was undertaken, as outcomes were reported in the original format used in the RCTs.

The identified interventions with animal-based foods or their extracts were described and presented in Table 4.

### 2.5. Data Synthesis and Analysis

In addition to outcome data, we extracted prespecified trial characteristics, including the animal-based food investigated and its formulation and dose (Table 4), as well as publication year, country, and population definition (MASLD alone or MASLD with cardiometabolic comorbidities such as type 2 diabetes mellitus, obesity, or metabolic syndrome). We recorded intervention and comparator details (dietary intervention description, type of control/placebo) and intervention duration. We also extracted baseline sample size overall and by study arm (intervention and control) and the analysis set reported by the trial authors (intention-to-treat (ITT), per-protocol (PP), or as-treated (AT)); if the analysis population was not specified, it was coded as “not reported”. Baseline participant characteristics included mean age (±SD) overall or by arm (as reported) and sex distribution (numbers of males and females, overall or by arm) (Table 5).

The collected data was analyzed and presented in a series of tables. The data analysis included comparison in respect to the category of metabolic outcomes and effect of intervention with an animal-based food or its extract, while comparing pre- and post-intervention value of the primary outcomes, including measures of (1) anthropometry, (2) lipid metabolism, and (3) liver function; and secondary outcomes of (4) glucose metabolism and (5) inflammatory status were presented.

The change reported in the metabolic outcomes following interventions with animal-based foods or their extracts was compared between the intervention and placebo/control groups and within each group with intervention. The *p*-value of less than 0.05 was considered significant when assessing the impact (the change) of the reported values of metabolic outcomes in the reviewed studies. Findings were interpreted and contrasted in the context of existing literature, discussing limitations, and providing implications for clinical practice and future research.

No meta-analysis was performed due to the limited evidence base (seven RCTs) and incomplete reporting of outcome data required to compute comparable between-group effect estimates (e.g., change scores and their variability). Most interventions were evaluated in only one trial, and substantial clinical and methodological diversity across studies (animal-based food type, dose, and follow-up duration) prevented quantitative pooling and a meaningful assessment of statistical heterogeneity. Consequently, formal exploration of heterogeneity (e.g., subgroup analyses or meta-regression), sensitivity analyses, and publication bias, such as funnel plots, and Egger’s test, was not undertaken. Accordingly, findings were synthesized and tabulated by outcome domain, with results structured by intervention type to facilitate descriptive comparisons across studies.

### 2.6. Risk of Bias Assessment

We conducted a comprehensive, standardized search across nine databases: PubMed, Scopus, Google Scholar, Cochrane CENTRAL, ClinicalTrials.gov, TRIPP, Web of Science, EMBASE, and EBSCO. Three independent reviewers performed the search and study selection using a priori inclusion and exclusion criteria, thereby minimizing selection bias and ensuring consistent eligibility. To harmonize the evidence base, only RCTs were included. All eligible records underwent independent critical appraisal by the three reviewers prior to data extraction. Data extraction followed a prespecified, standardized protocol to maximize comparability and reduce subjective interpretation. All prespecified metabolic endpoints reported by the included trials were analyzed to mitigate selective outcome reporting. For each study, we captured key methodological characteristics, including study design, sample size, participant age, intervention duration, features of the dietary intervention, and measured metabolic outcomes—thereby promoting standardized appraisal of study quality and limiting interpretative bias. Studies were additionally classified by statistical analysis strategy—intention-to-treat (ITT), per-protocol (PP), or as-treated (AT)—as detailed in Table 5. In ITT, all randomized participants are analyzed irrespective of protocol adherence; in PP, only participants who completed the intervention as assigned are included; and in AT, participants are analyzed according to the intervention actually received.

The risk of bias for each included study was assessed using the Cochrane Collaboration’s tool for assessing risk of bias (RoB 2.0). The risk bias assessment was conducted on 6 RCTs, as the results of one RCT were conducted on the same intervention.

Assessments were conducted across the following main bias domains: randomization process bias, intervention bias, data bias, measurement bias, and results selection bias. Each domain was evaluated and classified as “low risk,” “high risk,” or “some concerns.” There were 3 reviewers assessing the articles. Any discrepancies in risk of bias assessment were resolved through discussion or, if necessary, by consultation with an additional reviewer. To provide the inter-rater reliability metrics, the Krippendorf’s alpha scale was calculated with a result of 0.69, meaning good reliability.

**Figure 2:**
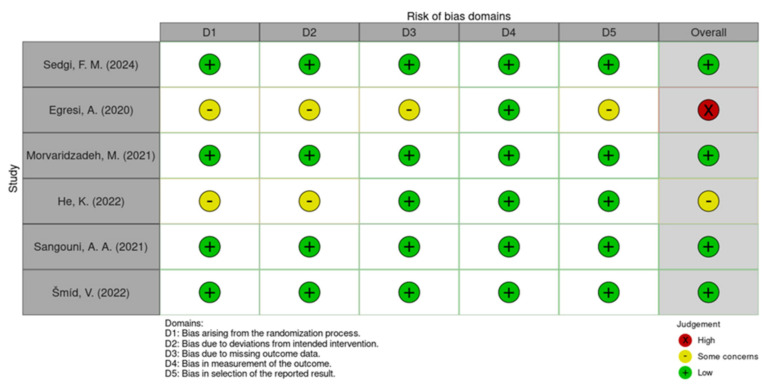
A graphical representation of the risk of bias domains [21,22,23,24,25,26,27].

**Figure 3:**
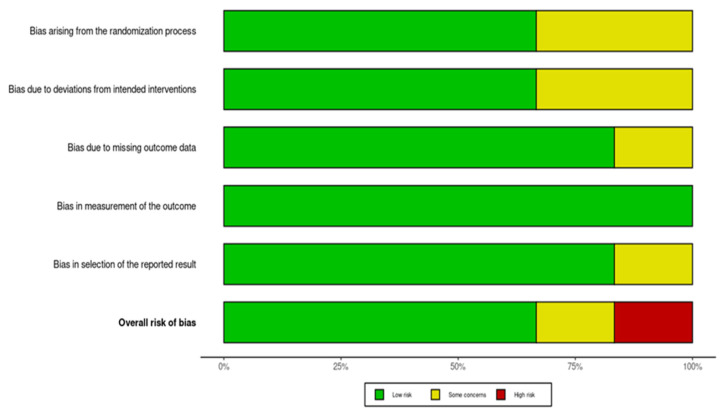
A graphical representation of the overall risk of bias of all RCTs.

A graphical representation of the risk of bias assessment results is provided in Figure 2 and Figure 3.

## 3. Results

### 3.1. Study Characteristics

Characteristics of foods of animal origin are included as part of the identified seven relevant RCT studies that are presented in Table 4 and Table 5. The selected RCTs investigated a range of animal-based foods, including dairy, fish, and fish-based oils, as well as red meat (Table 2). The type of intervention, its composition, and its duration varied among the studies. Most of the trials included interventions with fish and fish-based oils, including ~350 g daily intakes of freshwater fish, with bighead and grass carp as main sources of animal protein and fat [24]. Fish oils, including capsules containing omega-3, specifically EPA and DHA, were assessed in two trials [25,27], which varied in the dose and duration. The second most reviewed type of food in MASLD was dairy, such as yogurts whose composition varied from conventional, and bio (organic) to fortified with vitamin D versions [22,23]. Finally, animal-origin fat, in the form of ghee, was investigated in one study in which individuals with MASLD consumed this product as a part of their habitual diet with a frequency ranging from three to eight servings daily (equivalent to 15–40 g/day) [21].

All included RCTs in this review were published between 2020 and 2025 across diverse geographical regions, with most of these trials carried out in Iran and single studies in Hungary, China, and the Czech Republic (Table 2). All the included studies were conducted in adults who obtained a clinical diagnosis of MASLD, with the mean total age ranging between 40 and 52 years, and sample sizes from 34 to 88 participants considered in the analysis.

### 3.2. Influence of Dietary Interventions with Animal-Based Foods on Metabolic Outcomes in Patients with MASLD

The detailed values of the reported metabolic outcomes in identified RCTs are demonstrated in Tables 6, 8–10 and 12, and the comparisons of changes between animal-based foods and control/placebo are detailed in Tables 7, 11 and 13.

#### 3.2.1. Anthropometric Outcomes

The RCTs presenting effects of interventions with animal-based foods on anthropometric outcomes in patients with MASLD are presented in Table 6 and Table 7.

Among the reviewed RCTs, yogurt-based interventions, particularly with unfortified probiotic yogurt, were associated with reductions in body fat and blood pressure, and fish-based diets consistently improved central adiposity as reflected by decreased WC. (Table 6).

Among the clinical trials with animal-based foods on anthropometric measures among patients with MASLD, it was demonstrated that consumption of solely freshwater fish (*p* = 0.001), as well as eating both fresh fish and red meat (*p* = 0.022), can significantly reduce WC when compared with baseline. Nevertheless, these benefits were not observed with fish oil intake in the form of omega-3 supplementation with daily doses ranging from 2 to 3.6 g in both short-term (12 weeks) and long-term (48 weeks) when compared to baseline [25,26,27].

Interventions with dairy, although in the majority of studies have shown an insignificant effect on anthropometric measures, the trial assessing the impact of probiotic yogurt either fortified with vitamin D (*p* = 0.041) or unfortified (*p* = 0.008) demonstrated a significant decrease in systolic blood pressure (SBP) when compared to baseline values. Interestingly, solely unfortified probiotic yogurt [22]. Similar effects were observed following consumption of the unfortified probiotic yogurt, which significantly reduced BF percentage (*p* = 0.010) [23]. Nevertheless, none of these effects were observed following consumption of bio yogurt when compared to baseline measures [23]. Furthermore, substituting ghee as the main source of fat in the diet for 12 weeks had no significant impact on the BW and BMI; thus, the decreasing trend towards WC was reported when compared to the baseline values [21]. Although some results were statistically significant, the effect sizes were small and unlikely to be clinically meaningful, indicating only minimal improvements in outcomes.

The effects of animal-based dietary interventions on anthropometric outcomes in patients with MASLD were heterogeneous across the collected trials. Among the RCTs, exclusive consumption of probiotic yogurt fortified with vitamin D has significantly reduced BW (*p* = 0.047), with beneficial trends towards reduction in BMI, WC, BF, and blood pressure, when compared to intervention with unfortified probiotic yogurt [23]. The following tendency was reported for the intake of bio yogurt when compared to conventional yogurt; however, they remained insignificant [22]. Furthermore, freshwater fish consumption as the main source of protein and fat in the diet demonstrated the decreasing trend toward BW, BMI, and WC when compared to a diet with fish and meat eaten on alternate days [24]. A similar, thus insignificant, beneficial effect was observed with omega-3 oil supplementation, which has shown a decreasing tendency on the BW [25,26,27], as well as BMI and WC [27].

Solely eating ghee as a part of the usual diet had adverse effects on anthropometric measures, demonstrated as an increase in BW and BMI (*p* < 0.001 for all) when compared to partial substitution with plant-based fat [21].

#### 3.2.2. Glucose, Lipid Metabolism, and Inflammatory Outcomes

The RCT studies reporting impacts of intervention with animal-based foods on glucose, lipid, and inflammatory markers in patients with MASLD are presented in Table 8, Table 9 and Table 10, and the clinical significance of changes between the intervention and control/placebo is reported in Table 11.

The reviewed studies with animal-based foods in patients with MASLD showed heterogeneous effects on glucose homeostasis, lipid metabolism, and certain inflammatory markers.

Regarding glycemic management, the interventions with vitamin D-fortified probiotic yogurt led to significant improvements, including a reduction in fasting glucose (*p* < 0.001), insulin (*p* = 0.003), and HOMA-IR (*p* < 0.001), and these changes were clinically important [23]. A similar trend was also observed following intake of unfortified probiotic yogurt; however, these effects were not significant. Nevertheless, eating bio-yogurt and conventional yogurt has no significant impact on the glucose levels among patients with MASLD [22].

Daily consumption of freshwater fish as the main protein and fat source in the diet significantly reduced fasting glucose (*p* = 0.001) and triglycerides (*p* = 0.000) in contrast to a diet combining both fish with red meat, which attenuated this effect [24]. These changes were clinically important.

Interestingly, consuming ghee as a source of animal-based diet significantly increased fasting glucose (*p* = 0.008) and insulin (*p*< 0.001), whereas HOMA-IR significantly reduced (*p* < 0.001) [21] (Table 6).

According to the lipid profile (Table 9), the effects of animal-based foods assessed in the reviewed RCTs varied considerably across the interventions. A pronounced effect was observed with freshwater fish consumption, which reduced triglycerides (*p* < 0.001) and increased HDL-C (*p* = 0.005). When fish were combined with red meat, these benefits disappeared, and changes in TG and HDL-C were no longer statistically significant [24]. Omega-3 oil supplementation influenced cholesterol profile and reduced triglyceride levels; however, this effect was not significant when compared with baseline with post-intervention records [27]. Also, trials assessing the impact of bio- and conventional yogurts demonstrated no significant impact on lipid profile measures; consumption of bio-yogurt demonstrated a decreasing trend in triglycerides and cholesterol indices [22]. In contrast, ghee consumption increased concentrations of total cholesterol (results not significant) and LDL-C [21].

Furthermore, the inflammatory markers improved significantly following the daily intake of freshwater fish, demonstrated as lower IL-6 levels (*p* = 0.001), whereas the opposite, thus no significant effect, was reported in those who eat freshwater fish, with IL-6 levels even increasing slightly, though not significantly (*p* = 0.129) [24] (Table 10).

Comparison between RCTs with animal-based foods demonstrated mixed effects on metabolic and inflammatory outcomes in patients with MASLD (Table 11).

Among the studies, eating exclusively freshwater fish as the only source of protein and fat in the diet has shown the most prominent significant benefits for all indices of metabolic health, including significant reductions in fasting glucose (*p* = 0.002), triglycerides (*p* = 0.014), HDL-C (*p* = 0.004), and the inflammatory measure of IL-6 (*p* = 0.001) when compared to the intake of both fish and red meat [24]. Interestingly, intake of omega-3 oils, EPA, and DHA, when compared to placebo, led to significant reductions in triglycerides and HDL-C, whereas total cholesterol and LDL-C demonstrated an insignificant increasing trend [27].

Interventions assessing the impact of yogurts demonstrated a mixed, insignificant effect on metabolic outcomes. For example, eating probiotic yogurt fortified with vitamin D has improved glycemic control when compared to unfortified probiotic yogurt [23]. Similarly, consumption of bio yogurt obtained from organic farming significantly decreased fasting glucose, triglycerides, total cholesterol, and LDL-C (for all *p* > 0.05) when compared with conventional yogurt interventions [22].

Regular ghee intake led to significant increases in fasting glucose, insulin, and HOMA-IR (all *p* < 0.001), as well as an increase in total cholesterol (*p* = 0.006), compared with a diet in which ghee was replaced with plant-based oil [21] (Table 11).

#### 3.2.3. Liver Function Outcomes

The RCTs investigating the effects of animal-based foods on measures of liver, including hepatic enzymes, steatosis, and fibrosis, are presented in Table 12 and Table 13.

Among the studies, the most promising effects were observed following intervention with freshwater fish that significantly reduced ALT (*p* = 0.000) and also reduced the grade of fatty liver fat determined by Magnetic Resonance Imaging—Proton Density Fat Fraction (MRI-PDFF) (*p* = 0.000). Similar effect, thus insignificant, as also demonstrated for freshwater fish intake combined with red meat [24], and these results were clinically important. Interventions evaluating omega-3 effects also have beneficial effects on the hepatic enzymes, AST and ALT, as well as the fatty liver index (FLI); however, these effects remained insignificant when compared to placebo [27].

Significant and interesting benefits for hepatic enzymes were reported following consumption of ghee, which significantly reduced AST (*p* = 0.007) and ALP (*p* = 0.029), and an insignificant trend toward improvement in ALT. In addition, ghee intervention influenced the grade of fatty liver; as compared to baseline, the proportion of patients with grade 1 steatosis increased (0% to 18%), while grade 2 and 3 steatosis declined modestly [21]. However, although some changes reached statistical significance, their magnitude was small, and the clinical relevance remains uncertain. Contrastingly, both bio and conventional yogurts had increased hepatic enzymes; however, this effect was only significant for organic products and AST (*p* < 0.05) [22].

The comparison between RCTs with animal-based foods demonstrated limited effects on hepatic outcomes in patients with MASLD (Table 13).

Among the comparisons of selected RCTs, solely the intervention comparing the effect of freshwater fish consumption to a diet combining both fresh fish and red meat demonstrated a significant reduction in ALT (*p* = 0.002) and hepatic fat content measured by MRI-PDFF (*p* = 0.032) [24]. In regard to omega-3 obtained from fish oils, the benefits were reported in only one trial and were linked to insignificant improvement in hepatic enzymes, AST, ALT, grade of fatty liver, as well as fibrosis when compared with placebo [27].

For trials with dairy products, intake of bio yogurt, when compared to conventional yogurt, insignificantly decreased hepatic enzymes [22]. Ghee consumption, compared to replacement with plant-based oil, resulted in a significant reduction in ALP (*p* = 0.004); however, it had negative effects on the grade of fatty liver and AST and ALT, thus these observations remained insignificant [21].

## 4. Discussion

Nutritional modification is central to managing MASLD and preventing related metabolic complications [28]. Evidence consistently shows that the Mediterranean, DASH, and Nordic diets have the potential to improve anthropometric measures, lipid and glucose homeostasis, liver enzymes, and inflammation [7,28,29,30]. While benefits are often attributed to plant-based components, selected animal-derived foods, including fermented low-fat dairy, freshwater fish, and marine omega-3 oils, may also contribute positively to liver and metabolic health [15,16,31,32,33].

This systematic review presents RCT studies published from 2020 onward on the relationship between animal-based products and metabolic, anthropometric, and liver-related outcomes. However, the interpretation of these findings should be placed within the context of the pre-existing literature. Studies published before 2020 did not fulfill the predefined eligibility criteria for this systematic review and were therefore not included due to their high risk of bias.

Earlier evidence, including a meta-analysis published in 2020 [34] and based on pre-2020 studies, suggested that the association between specific food groups and NAFLD risk differed across animal-based products.

Red meat intake showed the most consistent positive association with NAFLD, whereas findings for fish, eggs, and dairy products were less consistent [34].

In particular, the significantly higher NAFLD risk was observed for red and processed meat, which may be partly explained by their saturated fat content, as saturated fat can promote hepatic lipid accumulation and insulin resistance. Additionally, heme iron may further impair insulin sensitivity through mechanisms related to oxidative stress. Processed meat may also contribute through its high sodium content and nitrite preservatives, which have likewise been linked to insulin resistance and hepatic steatosis [35].

The evidence for other animal-based foods is less consistent. Although some earlier data suggested that dairy products were not associated with NAFLD risk [34], a more recent meta-analysis showed an inverse association, indicating that dairy consumption may reduce the risk of NAFLD [16]. Similarly, fish consumption was not associated with NAFLD risk in a previous meta-analysis [34]; however, some individual studies reported an inverse association [36], but these findings should be interpreted with caution, as the genetic background of patients with NAFLD may influence the observed associations [37].

In the case–control study by Mokhtari et al., consuming 2–3 eggs per week was associated with approximately 3.7-fold higher odds of NAFLD compared with consuming fewer than 2 eggs per week [38]. In a later study published in 2020, higher egg consumption was associated with less favorable liver-related markers and a greater likelihood of NAFLD. Participants in the highest tertile of egg intake had 11% higher odds of NAFLD than those in the lowest tertile. However, after further adjustment for triglycerides, hypertension, and diabetes, this association was attenuated and became non-significant, suggesting that the observed relationship may have been largely mediated by coexisting cardiometabolic risk factors [39].

This updated systematic review provides new scientific evidence on the impact of animal-based foods on metabolic, anthropometric, and hepatic parameters in individuals who have developed MASLD.

Fermented low-fat dairy, such as kefir and probiotic yogurt, showed modest benefits; however, short times (8–12 weeks) and small amounts of products (100 g) could weaken the strength of these impacts [22,23]. Short-term trials reported higher HDL-Cl and lean mass without major changes in hepatic enzymes. However, the meta-analyses of probiotics overall indicate significant reductions in steatosis, liver enzymes, glucose, insulin, and lipids [40]. Mechanistically, these effects are mediated by modulation of the gut–liver axis, reducing endotoxemia and inflammation while enhancing fatty acid oxidation and insulin sensitivity [40]. This may attenuate NF-κB signaling and lower pro-inflammatory cytokine production in the liver. In addition, probiotic metabolites, like short-chain fatty acids (SCFAs), also improve hepatic fat oxidation and insulin sensitivity. The dairy matrix of probiotic beverages may also deliver nutrients like calcium and protein. However, the probiotics per se seem to play an important role in enhancing lipid metabolism and attenuating liver inflammation [40], thereby contributing to the modest improvements in liver health among patients with MASLD [23]. However, an important knowledge gap persists with respect to liver metabolism because most hepatic outcomes were not reported in the included studies.

In earlier studies not included in this systematic review, meat consumption has generally been associated with worse metabolic and hepatic parameters [41,42]. In the *Fatty Liver in Obesity* (FLiO) study of overweight/obese adults with ultrasound-confirmed NAFLD, higher red meat intake was associated with a less favorable liver-metabolic profile, including higher triglycerides, ALT, and ferritin concentrations, as well as lower HDL-cholesterol. Red and processed meat consumption were also positively associated with liver iron content, and for processed meat, this association remained significant after adjustment for potential confounders, like sex, age, body mass index, energy intake, and physical activity. Interestingly, no significant association was found between meat intake and liver fat content or HOMA-IR. In contrast, fish intake was inversely associated with ferritin concentration, suggesting a potentially more favorable hepatic profile [41].

This systematic review revealed that replacing red meat with fish improved MASLD outcomes independent of calorie restriction. In a controlled trial, a freshwater fish–based diet reduced glucose and hepatic fat and improved lipid profile more than a mixed fish–red meat diet, despite similar energy intake and weight stability [24]. However, the study design did not provide information on other cardiometabolic outcomes (blood pressure, insulin, HOMA-IR), which could further reveal important data. Fish provides high-quality protein, polyunsaturated fatty acids (PUFAs), and less saturated fat than red meat, with additional benefits from reduced heme iron and pro-inflammatory load [9,24,43,44]. Moreover, the gut microbiota shifts into increased activity of SCFA-producing genera (e.g., *Faecalibacterium*), followed by the higher production of fecal SCFAs, unconjugated bile acids, and reduced potentially pathogenic (*Prevotella* spp.) when compared with the mixed diet [24]. It may be considered a key driver of the positive effects attributed to the greater availability of essential amino acids, vitamin D, and taurine.

While higher intake of red and processed meat has been linked to less favorable hepatic and metabolic parameters, fish-derived omega-3 polyunsaturated fatty acids may exert beneficial effects on liver health. In a previous meta-analysis of RCTs published in 2020, omega-3 supplementation improved liver fat and was also associated with favorable changes in triglycerides, total cholesterol, HDL-cholesterol, and body mass index, although no clear benefit was observed for HOMA-IR or fasting blood glucose. These findings support the view that the type and nutritional quality of animal-based foods may be more relevant to NAFLD risk than animal-food intake per se [45]. However, more recent studies revealed that fish oil supplementation showed inconsistent results, including RCTs [25,26,27]. These results might be moderated by a small amount of biologically active omega-3 (EPA/DHA) in the presented RCTs. While presented studies reported no significant improvements in hepatic or metabolic indices, meta-analyses [12,46] suggest moderate benefits with high-dose (2–4 g/day EPA/DHA) regimens [40], including reduced liver fat, fibrosis progression, triglycerides, and improved insulin sensitivity. Anti-inflammatory effects, such as lower TNF-α levels, were also observed, although the magnitude of change was modest [40,47]. In contrast, high-saturated-fat animal products negatively influence metabolic parameters. A 12-week trial demonstrated worsened glycemic and lipid parameters but, interestingly, improved some hepatic outcomes in patients with MASLD consuming ghee. Replacing ghee with rapeseed oil significantly improved anthropometric measures, insulin sensitivity, and lipid profiles [21]. These findings underscore that high-SFA animal fats like ghee can exacerbate cardiometabolic outcomes, whereas shifting to unsaturated plant oils facilitates weight reduction, better lipid profiles, enhanced insulin sensitivity, and liver fat regression. Diets high in saturated fatty acids (e.g., ghee, butter) promote hepatic steatosis and inflammation via endoplasmic reticulum (ER)-stress–mediated activation of pro-inflammatory signaling and cytokine release, thereby aggravating insulin resistance. Replacing SFA with unsaturated fat, particularly MUFA-rich rapeseed oil (≈65% oleic acid) and PUFAs (≈40% linoleic, 10–15% α-linolenic acid) [48], favors β-oxidation, downregulates lipogenesis at the expense of upregulated fatty-acid catabolism, and attenuates inflammatory eicosanoid synthesis. All those activities collectively can improve hepatic lipid handling and insulin signaling [49]. Further randomized controlled trials assessing hepatic outcomes of SFA intake in patients with MASLD are warranted, given the inconclusive results presented [21].

This systematic review highlights several limitations across the recent trials. Specifically, the geographic concentration of studies in specific regions and the reliance on relatively small cohorts restrict the external validity and generalizability of the findings to wider, global MASLD populations. Furthermore, heterogeneous intervention designs, variable durations (4–48 weeks), and inconsistent dietary monitoring or quantification of bioactive compounds pose challenges to data synthesis. In addition, some data required for comprehensive clinical cardiometabolic assessments were missing in the reviewed studies, complicating the justification of the overall effect on hepatic and metabolic parameters. Due to these issues, this systematic review was conducted without a meta-analysis, as the lack of meaningful quantitative pooling precluded the approach proposed in the recent PRISMA 2020 guidance.

A further limitation concerns the evolving terminology from NAFLD to MASLD; while most included RCTs utilize the NAFLD framework, the MASLD definition incorporates explicit metabolic criteria. Although these definitions largely overlap, this transition may introduce minor differences in patient classification that should be considered when interpreting study populations. Nonetheless, this work possesses several strengths, including a focus on recent, high-quality RCTs with clinically relevant endpoints. Future research should adopt standardized intervention protocols, longer follow-up, and intention-to-treat designs to improve comparability and external validity. Notably, this review provides a detailed and evidence-based summary of the effects of animal-based food interventions on each clinically valid metabolic outcome in MASLD patients.

Given that earlier evidence on the effects of animal-based products on anthropometric, metabolic, and hepatic outcomes has already been summarized in meta-analyses, we restricted our review to the most recent period to avoid duplication. Accordingly, our conclusions primarily reflect the contemporary evidence base and may not encompass findings from earlier studies [50].

Overall, the evidence presented in this updated systematic review suggests that animal-source foods yield heterogeneous effects in MASLD, demonstrating that probiotic-enriched fermented dairy beverages and fish are generally beneficial, whereas fish-based omega-3 oils provide modest but consistent improvements in hepatic and cardiometabolic outcomes. Overall, regular consumption of saturated fats in the form of ghee may be metabolically unfavorable when it is not substituted with unsaturated plant oils. These findings support the integration of selected animal-based foods, such as fermented low-fat dairy and fish, within Mediterranean, DASH, and Nordic dietary patterns for patients with MASLD, while reinforcing the need to limit saturated fat–rich animal products. From a clinical perspective, emphasis should be placed on whole-food sources rather than isolated supplements, as benefits appear more consistent for food matrices than for omega-3 oils at commonly used doses. Future randomized controlled trials should adopt standardized dietary protocols, clearly quantify bioactive components, and include longer follow-up periods with clinically relevant hepatic and cardiometabolic endpoints. In particular, structured outcome reporting and intention-to-treat analyses are needed to improve comparability across studies and enable robust quantitative synthesis. Such methodological improvements will be essential to strengthen evidence-based dietary recommendations for MASLD management.

## 5. Conclusions

The main conclusions of this review indicate that certain whole animal-derived foods can play a potential supportive role in the dietary management of patients with MASLD when consumed as part of the MED/DASH-style pattern.

Regular consumption of freshwater fish as a primary protein and fat source has been associated with improvements in glycemic control, lipid profiles, inflammatory markers, and selected hepatic outcomes. Daily intake of probiotic yogurt, particularly if fortified with vitamin D, may offer favorable effects on glycemic regulation, adiposity, and blood pressure in patients with MASLD. Supplementation with fish oil as a source of DHA and EPA may offer potential improvements in lipid parameters, liver steatosis, fibrosis markers, and anthropometric and enzymatic outcomes; however, given the heterogeneity in dosing strategies and intervention durations among limited trails this needs to be further investigated. Therefore, the key clinically relevant takeaway of this review is to consider incorporation of fish and fortified probiotic dairy as components of nutrition plans for MASLD while limiting foods high in saturated fat until stronger evidence becomes available. As the number of trials remains small and study designs vary considerably, further well-designed RCTs are needed to clarify the role of specific animal-based foods in MASLD management and refine practical dietary recommendations. Further randomized controlled trials with standardized protocols are needed to clarify the role of animal-based foods in the diets of patients with MASLD and those at high risk.

## Figures and Tables

**Table 1:** PICO table to determine the eligibility of the research question.

Criteria	Determinants
Population (P)	Adults aged 18–65 years diagnosed with MASLD using validated methods such as liver biopsy or ultrasonography, with or without comorbidities including obesity, metabolic syndrome, type 2 diabetes, or insulin resistance.
Intervention (I)	Dietary intake of single animal-based foods, food groups, or powdered forms, including fish, poultry, red meat, dairy, eggs, fermented products, and protein supplements.
Comparison (C)	Control diets, standard care, or alternative dietary interventions not emphasizing animal-based foods.
Outcome (O)	Primary: metabolic and hepatic measures, including anthropometric parameters (body weight, BMI, waist circumference, and body fat), lipid profile (triglycerides, total cholesterol, LDL, and HDL), hepatic enzymes (ALT, AST, and ALP), and liver fat/steatosis and fibrosis indices (CAP, FLI, fatty liver grade, and Fibroscan). Secondary: glucose metabolism (fasting glucose, insulin, HbA1c, and HOMA-IR), and inflammatory markers (hs-CRP and LPS)
Study design (S)	RCTs published between 1 January 2020 and 31 March 2026.

**Table 2:** Search strategy in the databases: terms, algorithms, and search blocks.

**Database**	PubMed (National Library of Medicine), Scopus (Elsevier), Embase via platform Embase.com, Web of Science Core Collection (Clarivate), the Cochrane Central Register of Controlled Trials (CENTRAL) via the Cochrane Library, CINAHL via EBSCOhost, Google Scholar, ClinicalTrials.gov (https://clinicaltrials.gov/), the TRIP Database (https://www.tripdatabase.com/).
**Liver disease and comorbidities terms**	“NAFLD”, “Non-alcoholic Fatty Liver Disease”, “MASLD”, “Metabolic Dysfunction-Associated Steatotic Liver Disease”, “obesity”, “type 2 diabetes”, “diabetes mellitus”, “metabolic syndrome”
**Animal-based product terms**	“Anchovies” OR “Salmon” OR “Sardines” OR “Mackerel” OR “Tuna” OR “Herring” OR “Cod” OR “ Pollock” OR “Flounder” OR “Whitefish” OR “Tilapia” OR “Sea bass” OR “Sea bream” OR “Fatty fish” OR “White fish” OR “Fish oils” OR “Caviar” OR “Seafood” OR “Mussels” OR “Clams” OR “Shrimp” OR “Prawns” OR “Scallops” OR “Oysters” OR “Crab” OR “Lobster” OR “Fish Meat” OR “Chicken” OR “Turkey” OR “Duck” OR “Rabbit” OR “Poultry” OR “Game” OR “Beef” OR “Lean beef cuts (tenderloin, sirloin, round)” OR “Lamb” OR “Pork” OR “Lean pork cuts (tenderloin, loin chop)” OR “Goat” OR “Red meat” OR “Ham” OR “Milk” OR “Dairy” OR “Cow Milk” OR “Goat Milk” OR “Sheep Milk” OR “Full-fat Milk” OR “Low-fat Milk” OR “Skimmed Milk” OR “Yogurt” OR “Regular yogurt” OR “Greek yogurt” OR “Plain yogurt” OR “Low-fat yogurt” OR “Fat-free yogurt” OR “Low-sugar yogurt” OR “Kefir” OR “Ayran” OR “Labneh” OR “Skyr” OR “Quark” OR “Cottage cheese” OR “Cream” OR “Butter” OR “Ghee” OR “Buttermilk” OR “Fermented milk drink” OR “Fermented foods” OR “Cheese” OR “Feta” OR “Mozzarella” OR “Mascarpone” OR “Ricotta” OR “Gouda” OR “Parmesan” OR “Parmigiano-Reggiano” OR “Hard cheese” OR “Pecorino” OR “Manchego” OR “Soft cheese” OR “Chevre” OR “Goat cheese” OR “Animal -based food” OR “Egg” OR “Mayonnaise” OR “Honey” OR “Propolis” OR “Royal jelly” OR “Whey proteins” OR “Protein powders” OR “Collagen” OR “Gelatin” OR “Omega-3” OR “DHA” OR “EPA” OR “Grass-fed” OR “Lard”.
**Terms algorithm**	Pairing each liver disease term with the dietary keyword and with major metabolic comorbidities relevant to MASLD pathophysiology.
**Search blocks**	Example: “MASLD AND food,” “MASLD AND obesity AND food,” “MASLD AND metabolic syndrome AND food,” “MASLD AND diabetes type 2 AND food,” Equivalent combinations for NAFLD and for the full spelled-out terms. The same approach was applied for “Metabolically Dysfunction-Associated Steatotic Liver Disease” and “Non-Alcoholic Fatty Liver Disease” to ensure that studies indexed under older or newer nomenclature were equally captured.

**Table 3:** Detailed rationale for specific exclusion criteria.

Exclusion	Reason
Participants aged <18 years and >65 years	Children, older adults, and pregnant women were excluded because of important differences in physiology, nutritional needs, and metabolic responses.
Studies other than RCTs	RCTs provide the highest level of evidence for evaluating the effects of dietary interventions and reduce the risk of confounding inherent in observational designs.
RCTs published earlier than 1 January 2020 and later than 31 March 2026	Earlier evidence on NAFLD as well as key metabolic outcomes has already been comprehensively synthesized in prior systematic reviews and meta-analyses. This time restriction was therefore introduced to avoid unnecessary duplication of previously summarized evidence and to provide an up-to-date synthesis aligned with the current transition from NAFLD to MASLD terminology and contemporary clinical research priorities.
Studies evaluated: – dietary supplements, medications, or plant-based products; – caloric restrictions; – nutritional education; – combined dietary interventions with broader lifestyle modifications (e.g., behavioral or cognitive-behavioral therapies, psychoeducational programs, self-management strategies, motivational interviewing, physical activity, sleep hygiene, stress management, or changes in diet quality and habits).	Such multicomponent approaches would make it difficult to attribute observed effects specifically to the investigated animal-based food intervention. Studies combining diet with broader lifestyle or behavioral interventions were also excluded to minimize confounding and allow attribution of outcomes to the dietary intervention itself.

**Table 4:** Animal-based foods included in the reviewed RCTs, along with their form/examples used in the intervention.

Ref	Name of Animal-Based Food	Form/Examples	Dose	Duration	Additional Comments
[21]	Animal-based fat	Ghee	Intake from three to eight servings of ghee daily, and 5 g of ghee is one serving.	12 weeks	N/A
[22]	Yogurt	Bio yogurt	300 g serving a day	8 weeks	Bio (organic) yogurt was enriched with calcium, phosphorus, iron, and zinc; in addition to 2.4 × 10^8^ CFU of probiotic bacteria, such as Streptococcus thermophilus and Lactobacillus bulgaricus. This yogurt provides 68 kcal/100 g along with 4.2 g of protein, 4.2 g of carbohydrates, and 3.6 g of fat; and minimal amounts of vitamins D and E, magnesium, selenium, copper, and manganese.
[22]		Conventional yogurt	300 g serving a day	8 weeks	The conventional yogurt has 3.0 × 10^8^ CFU of probiotic bacteria, including Streptococcus thermophilus and Lactobacillus bulgaricus, and provides 65 kcal/100 g, with 3.4 g protein, 4.8 g carbohydrates, and 3.6 g fat, including 2.4 g saturated fat. It contains modest amounts of calcium, phosphorus, iron, and zinc.
[23]		Probiotic yogurt fortified with vitamin D	100 g serving a day	12 weeks	Probiotic yogurt with a fat content of less than 1% and 4 × 10^7^ CFU of probiotic bacteria enriched with Lactobacillus acidophilus La-5 and Bifidobacterium lactis Bb-12, and enriched with 1000 IU of vitamin D.
[23]		Unfortified probiotic yogurt	100 g serving a day	12 weeks	Probiotic yogurt with a fat content of less than 1% and 4 × 10^7^ CFU of probiotic bacteria Streptococcus thermophilus and Lactobacillus bulgaricus.
[24]	Fish	Freshwater Fish (i.e., alternation of bighead carp and grass carp on a daily basis)	The daily freshwater fish intake was on average 344.12 g (SD 63.65).	84 days	Intake of only freshwater fish (i.e., alternation of bighead carp and grass carp on a daily basis) as the only source of animal protein and fat in the usual diet.
[24]	Fish and red meat	Fish (i.e., bighead and grass carp) and red meat (i.e., beef, pork, and mutton)	The daily freshwater fish consumption was on average 143.24 (SD 16.48), and red meat was 143.24 g (SD 23.91).	12 weeks (84 days)	A combination of freshwater fish (i.e., bighead and grass carp) and red meat (i.e., beef, pork, and mutton) with a daily alternation (namely, 1 cycle of this alternate diet is a 2-day period) served as the only source of animal protein and fat in the usual diet
[25]	Fish Oil	Omega-3, EPA & DHA	Daily intake of 2 g of Omega-3 in the form of 2 gel capsules, as each capsule (1000 mg) contained 180 mg EPA and 120 mg DHA).	12 weeks	N/A
[26]					
[27]			Daily intake of total 3.6 g of Omega-3, given in the tablets (2 tables, 4 times a day, as each table contained 450 mg of Omega-3, in the form of EPA (215 mg, 48%) and DHA (155 mg, 34%).	48 weeks (12 months)	

**Table 5:** Main characteristics of the RCT studies included in the review.

Ref	Year	Country	Population	Number of Participants at the Baseline	Age (Mean ± SD) at the Baseline	Sex (n)	Dietary Intervention with Animal-Based Food				Type of Analysis
							Dietary Intervention	Duration	Intervention Group	Control/Placebo	ITT, PP or AT
[21]	2024	Iran	MASLD	TOTAL: 60 I: 30; C: 30	TOTAL: 42 ± 9.6	I: M 35, F 20 C: M 35, F 20	**Ghee**	12 weeks	Ghee was substituted with rapeseed oil in the same amount as part of the diet.	CONTROL: continued the consumption of ghee as part of a healthy diet.	PP
[22]	2020	Hungary	MASLD with T2DM	TOTAL: 37 I: 21; C: 16	TOTAL: 51.73 ± 11.82	M 21, F 16	**Yogurt**	8 weeks	Intake of bio yogurt from organic farming.	CONTROL: intake of conventional yogurt.	not reported
[24]	2022	China	MASLD	TOTAL: 34 I: 17; C: 17	I: 48.00 ± 12.71; C: 43.59 ± 12.29	N/A	**Freshwater fish**	12 weeks (84 days)	Freshwater fish as the main source of protein; bighead carp and grass carp consumed on a daily basis.	CONTROL: intake of protein in the alternate daily manner from both freshwater fish (i.e., bighead and grass carp) and red meat (i.e., beef, pork, and mutton)	ITT
[23]	2021	Iran	MASLD	TOTAL: 88 I: 44; C: 44	I: 40.39 ± 6.22; C: 39.91 ± 7.16	M 47, F 41	**Probiotic yogurt**	12 weeks	Daily consumption of a 100 g serving of probiotic yogurt fortified with vitamin D	CONTROL: daily intake of 100 g serving of unfortified probiotic yogurt.	PP
[25]	2021	Iran	MASLD with T2DM and obesity	TOTAL: 56 I: 28; C: 28	I: 48.6 ± 7.6; C: 48.8 ± 8.7	I: M 9, F 21 C: M 6, F 24	**Omega-3 oil (EPA and DHA)**	12 weeks	Daily intake of two gel capsules containing Omega-3 (each capsule was 1000 mg and provided 180 mg EPA and 120 mg DHA).	CONTROL: PLACEBO—daily intake of 2 g placebo in the form of a gel capsule, which contains liquid paraffin.	ITT
[26]											
[27]	2022	Czech Republic	MASLD with MS	TOTAL: 60 I: 30; C: 30	I: 51.8 ± 12; C: 52.1 ± 12	I: M 21, F 9 C: M 24, F 6		48 weeks (12 months)	Daily intake of a total of 3.6 g of Omega-3, given in the tablets (2 tablets, 4 times a day, as each tablet contained 450 mg of Omega-3, in the form of EPA (215 mg, 48%) and DHA (155 mg, 34%).	CONTROL: PLACEBO—daily intake of a total of 3.6 g of placebo (2 tablets, 4 times a day, as each tablet contained 450 mg of soya oil).	PP

ITT, intention to treat; PP, per protocol; AT, as treated; MASLD, metabolic dysfunction-associated steatotic liver disease; previously termed NAFLD; T2DM, type 2 diabetes mellitus; MS, metabolic syndrome; I, intervention; C, control; M, male; F, female; N/A, states where there was no data available. Publication bias was not formally assessed using funnel plots or some other statistical tests because the number of included RCT studies was insufficient (n < 10) to provide reliable results, as per Cochrane guidelines.

**Table 6:** Anthropometric outcomes upon intervention with animal-based foods in the RCTs conducted in patients with MASLD.

Dietary Intervention		Anthropometric Outcomes After Intervention with Animal-Based Foods																	
		BW			BMI			WC			BF			DBP			SBP		
		B	PI	*p*	B	PI	*p*	B	PI	*p*	B	PI	*p*	B	PI	*p*	B	PI	*p*
[21]	**Ghee**	81.7 ± 7.6	81.7 ± 7.2	*p* = 0.674	28.23 ± 1.5	28.3 ± 1.6	*p* = 0.645	N/A	N/A	N/A	N/A	N/A	N/A	N/A	N/A	N/A	N/A	N/A	N/A
[22]	**Bio Yogurt**	89.0 ± 15.5	88.59 ± 15.38	NS	31.61 ± 5.4	31.46 ± 5.31	NS	168.0 ± 10.08	168.14 ± 10.03	NS	34.91 ± 10.05	35.38 ± 9.73	NS	N/A	N/A	N/A	N/A	N/A	N/A
[22]	**Conventional Yogurt**	N/A	N/A	N/A	N/A	N/A	N/A	N/A	N/A	N/A	N/A	N/A	N/A	N/A	N/A	N/A	N/A	N/A	N/A
[23]	**Probiotic yogurt fortified with vitamin D**	79.59 ± 9.60	79.21 ± 9.49	*p* = 0.254	28.38 ± 3.09	28.23 ± 2.94	*p* = 0.191	96.11 ± 9.05	95.21 ± 8.79	*p* = 0.267	24.49 ± 5.96	23.88 ± 5.09	*p* = 0.058	78.82 ± 8.74	77.07 ± 8.71	*p* = 0.234	121.14 ± 13.58	117.73 ± 12.20	*p* = 0.041
[23]	**Unfortified probiotic yogurt**	81.24 ± 12.59	80.91 ± 12.59	*p* = 0.216	28.45 ± 2.43	28.34 ± 2.49	*p* = 0.238	96.13 ± 7.96	96.59 ± 7.52	*p* = 0.535	25.24 ± 5.11	24.62 ± 5.24	*p* = 0.010	79.91 ± 11.22	78.36 ± 8.53	*p* = 0.330	121.50 ± 12.10	116.34 ± 11.48	*p* = 0.008
[24]	**Freshwater Fish**	72.50 (67.65, 87.05)	72.00 (66.95, 87.30)	*p* = 0.123	28.72 ± 3.82	28.18 ± 3.70	*p* = 0.093	97.18 ± 9.95	93.59 ± 9.31	*p* = 0.001	N/A	N/A	N/A	N/A	N/A	N/A	N/A	N/A	N/A
[24]	**Freshwater Fish and red meat**	79.50 (74.00, 92.50)	79.50 (73.00, 92.75)	*p* = 0.086	28.76 ± 4.58	28.44 ± 4.60	*p* = 0.063	99.18 ± 10.77	96.47 ± 10.93	*p* = 0.022	N/A	N/A	N/A	N/A	N/A	N/A	N/A	N/A	N/A
[25]	**Omega-3 oil**	78.0 ± 10.5	78.0 ± 11.1	*p* = 0.710	30.26 ± 3.6	30.23 ± 3.7	*p* = 0.590	106.7 ± 9.1	106.1 ± 8.2	*p* = 0.590	N/A	N/A	N/A	N/A	N/A	N/A	N/A	N/A	N/A
[26]		78.0 ± 10.5	78.0 ± 11.1	*p* = 0.710	30.26 ± 3.6	30.23 ± 3.7	*p* = 0.590	106.7 ± 9.1	106.1 ± 8.2	*p* = 0.590	N/A	N/A	N/A	N/A	N/A	N/A	N/A	N/A	N/A
[27]		96.2 ± 16.7	94.7 ± 15.2	NS	30.0 ± 3.3	30.8 ± 4.9	NS	106.6 ± 8.8	105.3 ± 9.0	NS	N/A	N/A	N/A	N/A	N/A	N/A	N/A	N/A	N/A

Data is presented as Mean ± SD, or Median (25th–75th). Reported B and PI as exact values. N/A states that there was no data available. BW expressed in kilograms (kg). BMI expressed in kg/m^2^. WC expressed in cm. SBP and DBP are expressed as mmHg. *p* value > 0.05 results considered as NS. BW, Body Weight; BMI, Body Mass Index; WC, Waist Circumference; BF, Body Fat; DBP, Diastolic Blood Pressure; SBP, Systolic Blood Pressure; SD, Standard Deviation; NS, Not Significant; N/A, states where there was no data available; B, Baseline; PI, Post-intervention.Comparison between RCTs with animal-based foods demonstrated mixed effects on metabolic and inflammatory outcomes in patients with MASLD (Table 7).

**Table 7:** Comparisons of changes in anthropometric outcomes after interventions with animal-based foods and control/placebo in the RCTs conducted in patients with MASLD.

Change in the Anthropometric Outcomes Following Interventions with Animal-Based Foods in Patients with MASLD							
Reference	Intervention	BW	BMI	WC	BF	DBP	SBP
[21]	**Ghee**	*p* < 0.001	*p* < 0.001	N/A	N/A	N/A	N/A
[22]	**Bio Yogurt**	*p* > 0.05	*p* > 0.05	*p* > 0.05	*p* > 0.05	N/A	N/A
[22]	**Conventional Yogurt**	*p* > 0.05	*p* > 0.05	*p* > 0.05	*p* > 0.05	N/A	N/A
[23]	**Probiotic yogurt fortified with vitamin D**	*p* = 0.047	*p* = 0.840	*p* = 0.430	*p* = 0.500	*p* = 0.480	*p* = 0.580
[23]	**Unfortified probiotic yogurt**	*p* = 0.047	*p* = 0.840	*p* = 0.430	*p* = 0.500	*p* = 0.480	*p* = 0.580
[24]	**Freshwater Fish**	*p* = 0.683	*p* = 0.551	*p* = 0.530	N/A	N/A	N/A
[24]	**Freshwater Fish and red meat**	*p* = 0.683	*p* = 0.530	*p* = 0.530	N/A	N/A	N/A
[25]	**Omega-3 oil**	*p* = 0.610	*p* = 0.560	*p* = 0.270	N/A	N/A	N/A
[26]		*p* = 0.610	*p* = 0.560	*p* = 0.270	N/A	N/A	N/A
[27]		*p* > 0.05	*p* > 0.05	*p* > 0.05	N/A	N/A	N/A

For each comparison, we report the *p* value and the direction of change in PI. Cell background color indicates statistical significance—green for significant results (e.g., *p* < 0.05) and red for non-significant results (*p* ≥ 0.05). Independently, the color of the numeric value (value text) reflects effect direction: green denotes a positive (favorable) change in PI, whereas red denotes a negative (unfavorable) change. N/A, states where there was no data available. BW, Body Weight; BMI, Body Mass Index; WC, Waist Circumference; BF, Body Fat; DBP, Diastolic Blood Pressure; SBP, Systolic Blood Pressure.

**Table 8:** Outcomes of glucose metabolism upon intervention with animal-based foods in the RCTs conducted in patients with MASLD.

Dietary Intervention		Glucose Metabolism Outcomes After Intervention with Animal-Based Foods											
		Glucose			Insulin			HOMA-IR			HbA1c		
		B	PI	*p*	B	PI	*p*	B	PI	*p*	B	PI	*p*
[21]	**Ghee**	96.7 ± 11.6	99.5 ± 13.6	*p* = 0.996	12.6 ± 4.9	17.5 ± 5.7	*p* = 0.622	3.00 ± 1.3	4.4 ± 1.7	*p* = 0.645	N/A	N/A	N/A
[22]	**Bio Yogurt**	5.59 ± 1.41	5.92 ± 1.37	NS	N/A	N/A	N/A	N/A	N/A	N/A	N/A	N/A	N/A
[22]	**Conventional Yogurt**	5.63 ± 1.34	5.99 ± 1.64	NS	N/A	N/A	N/A	N/A	N/A	N/A	N/A	N/A	N/A
[23]	**Probiotic Yogurt Fortified with Vitamin D**	102.29 ± 10.59	90.36 ± 9.66	*p* < 0.001	17.56 ± 10.04	13.40 ± 5.70	*p* = 0.003	4.51 ± 2.65	2.98 ± 1.24	*p* < 0.001	N/A	N/A	N/A
[23]	**Unfortified Probiotic Yogurt**	100.04 ± 12.16	91.54 ± 15.74	*p* < 0.001	15.69 ± 9.35	13.17 ± 5.01	*p* = 0.060	3.94 ± 2.53	3.01 ± 1.37	*p* = 0.13	N/A	N/A	N/A
[24]	**Freshwater Fish**	5.57 (5.11, 5.81)	5.02 (4.55, 5.61)	*p* = 0.001	N/A	N/A	N/A	4.47 ± 1.81	N/A	N/A	N/A	N/A	N/A
[24]	**Freshwater Fish and Red Meat**	5.34 (5.11, 6.19)	5.54 (5.10, 6.42)	*p* = 0.397	N/A	N/A	N/A	4.97 ± 2.96	N/A	N/A	N/A	N/A	N/A
[25]	**Omega-3 Oil**	N/A	N/A	N/A	N/A	N/A	N/A	N/A	N/A	N/A	N/A	N/A	N/A
[26]		N/A	N/A	N/A	N/A	N/A	N/A	N/A	N/A	N/A	N/A	N/A	N/A
[27]		N/A	N/A	N/A	N/A	N/A	N/A	N/A	N/A	N/A	41.8 ± 9.1	45.8 ± 13.3	NS

Data is presented as Mean ± SD, or Median (25th–75th). N/A states that there was no data available. Glucose expressed in mg/dL. Insulin expressed in µU/mL. HbA1c expressed in %. *p* value > 0.05 results considered as NS. HOMA-IR, Homeostatic Model Assessment of Insulin Resistance; HbA1c, Hemoglobin A1c; SD, Standard Deviation; NS, Not Significant; N/A, states where there was no data available; B, Baseline; PI, Post-intervention.

**Table 9:** Outcomes of lipid metabolism upon intervention with animal-based foods in the RCTs conducted in patients with MASLD.

Dietary Intervention		Lipid Metabolism Outcomes After Intervention with Animal-Based Foods											
		Triglycerides			Cholesterol			LDL-C			HDL-C		
		B	PI	*p*	B	PI	*p*	B	PI	*p*	B	PI	*p*
[21]	**Ghee**	N/A	N/A	N/A	191.5 ± 43.7	195.4 ± 41.3	*p* = 0.264	109.8 ± 30.6	116.3 ± 28.9	*p* = 0.032	N/A	N/A	N/A
[22]	**Bio Yogurt**	1.86 ± 0.73	1.61 ± 0.67	NS	5.59 ± 0.85	5.63 ± 0.87	NS	64.34 ± 3.27	63.13 ± 2.97	NS	23.91 ± 5.4	23.88 ± 4.48	NS
[22]	**Conventional Yogurt**	1.84 ± 0.75	1.99 ± 0.88	NS	5.68 ± 1.17	5.77 ± 1.31	NS	64.71 ± 4.68	64.06 ± 3.1	NS	25.97 ± 5.6	23.1 ± 4.35	NS
[23]	**Probiotic Yogurt Fortified with Vitamin D**	N/A	N/A	N/A	N/A	N/A	N/A	N/A	N/A	N/A	N/A	N/A	N/A
[23]	**Unfortified Probiotic Yogurt**	N/A	N/A	N/A	N/A	N/A	N/A	N/A	N/A	N/A	N/A	N/A	N/A
[24]	**Freshwater Fish**	2.63 ± 0.63	1.76 ± 0.58	*p* = 0.000	5.29 (4.50, 6.33)	N/A	N/A	3.47 (2.68, 4.33)	N/A	N/A	1.19 ± 0.26	1.27 ± 0.27	*p* = 0.005
[24]	**Freshwater Fish and Red Meat**	2.32 ± 1.11	2.03 ± 1.01	*p* = 0.147	4.98 (4.44, 5.48)	N/A	N/A	3.23 (2.74, 3.81)	N/A	N/A	1.13 ± 0.13	1.10 ± 0.16	*p* = 0.283
[25]	**Omega-3 oil**	151.6 ± 57.5	N/A	N/A	N/A	N/A	N/A	N/A	N/A	N/A	45.3 ± 8.9	N/A	N/A
[26]		151.6 ± 57.5	N/A	N/A	166.0 ± 41.1	N/A	N/A	95.9 ± 30.0	N/A	N/A	45.3 ± 8.9	N/A	N/A
[27]		2.08 ± 1.6	2.03 ± 1.5	NS	5.06 ± 1.2	5.21 ± 1.0	NS	2.91 ± 1.1	3.14 ± 1.1	NS	1.32 ± 0.3	1.21 ± 0.3	NS

Data is presented as Mean ± SD, or Median (25th–75th). N/A states that there was no data available. Triglycerides, cholesterol, LDL-C, and HDL-C expressed in mg/dL. *p* value > 0.05 results are considered as NS. HDL-C, High-Density Lipoprotein Cholesterol; LDL-C, Low-Density Lipoprotein Cholesterol; SD, Standard Deviation; NS, Not Significant; N/A, states where there was no data available; B, Baseline; PI, Post-intervention.

**Table 10:** Outcomes of inflammatory metabolism upon intervention with animal-based foods in the RCTs conducted in patients with MASLD.

Dietary Intervention		Inflammatory Outcomes After Intervention with Animal-Based Foods					
		hs-CRP			IL-6 (pg/mL)		
		B	PI	*p*	B	PI	*p*
[21]	**Ghee**	N/A	N/A	N/A	N/A	N/A	N/A
[22]	**Bio Yogurt**	N/A	N/A	N/A	N/A	N/A	N/A
[22]	**Conventional Yogurt**	N/A	N/A	N/A	N/A	N/A	N/A
[23]	**Probiotic Yogurt Fortified with Vitamin D**	N/A	N/A	N/A	N/A	N/A	N/A
[23]	**Unfortified Probiotic Yogurt**	N/A	N/A	N/A	N/A	N/A	N/A
[24]	**Freshwater Fish**	2.20 (1.15, 4.30)	N/A	N/A	2.77 ± 0.62	2.20 ± 0.38	*p* = 0.001
[24]	**Freshwater Fish and Red Meat**	(1.15, 4.30) 1.90 (1.00, 2.95)	N/A	N/A	2.73 ± 0.82	2.99 ± 1.09	*p* = 0.129
[25]	**Omega-3 oil**	N/A	N/A	N/A	N/A	N/A	N/A
[26]		N/A	N/A	N/A	N/A	N/A	N/A
[27]		N/A	N/A	N/A	N/A	N/A	N/A

Data is presented as Mean ± SD, or Median (25th–75th). N/A states that there was no data available. hs-CRP expressed in ng/mL. IL-6 expressed in pg/mL. *p* value > 0.05 results considered as NS. IL-6, Interleukin-6; hs-CRP, High-Sensitivity C-Reactive Protein; SD, Standard Deviation; NS, Not Significant; N/A, states where there was no data available; PI, Post-Intervention.

**Table 11:** Comparisons of changes in glucose, lipid, and inflammatory outcomes after interventions with animal-based foods and control/placebo in the RCTs conducted in patients with MASLD.

Change in the Glucose and Lipid Metabolism Outcomes Along with Inflammatory Status Between Interventions with Animal-Based Foods in MASLD											
Reference	Intervention	Glucose	Insulin	HOMA-IR	HbA1c	Triglycerides	Cholesterol	LDL-C	HDL-C	hs-CRP	IL-6
[21]	**Ghee**	*p* < 0.001	*p* < 0.001	*p* < 0.001	N/A	N/A	*p* = 0.006	*p* = 0.07	N/A	N/A	N/A
[22]	**Bio Yogurt**	*p* > 0.05	N/A	N/A	N/A	*p* > 0.05	*p* > 0.05	*p* > 0.05	*p* > 0.05	N/A	N/A
[22]	**Conventional Yogurt**	*p* > 0.05	N/A	N/A	N/A	*p* > 0.05	*p* > 0.05	*p* > 0.05	*p* > 0.05	N/A	N/A
[23]	**Probiotic Yogurt Fortified with Vitamin D**	*p* = 0.910	*p* = 0.840	*p* = 0.92	N/A	N/A	N/A	N/A	N/A	N/A	N/A
[23]	**Unfortified Probiotic Yogurt**	*p* = 0.910	*p* = 0.840	*p* = 0.92	N/A	N/A	N/A	N/A	N/A	N/A	N/A
[24]	**Freshwater Fish**	*p* = 0.002	N/A	N/A	N/A	*p* = 0.014	N/A	N/A	*p* = 0.004	N/A	*p* = 0.001
[24]	**Freshwater Fish and Red Meat**	*p* = 0.002	N/A	N/A	N/A	*p* = 0.014	N/A	N/A	*p* = 0.004	N/A	*p* = 0.001
[25]	**Omega-3 Oil**	N/A	N/A	N/A	N/A	N/A	N/A	N/A	N/A	N/A	N/A
[26]		N/A	N/A	N/A	N/A	N/A	N/A	N/A	N/A	N/A	N/A
[27]		N/A	N/A	N/A	N/A	*p* > 0.05	*p* > 0.05	*p* > 0.05	*p* > 0.05	N/A	N/A

For each comparison, we report the *p* value and the direction of change in PI. Cell background color indicates statistical significance—green for significant results (e.g., *p* < 0.05) and red for non-significant results (*p* ≥ 0.05). Independently, the color of the numeric value (value text) reflects effect direction: green denotes a positive (favorable) change in PI, whereas red denotes a negative (unfavorable) change N/A states that there was no data available. *p* value > 0.05 results considered as NS. HOMA-IR, Homeostatic Model Assessment of Insulin Resistance; HbA1c, Hemoglobin A1c; HDL-C, High-Density Lipoprotein Cholesterol; LDL-C, Low-Density Lipoprotein Cholesterol; CRP, High-Sensitivity C-Reactive Protein.

**Table 12:** Liver function upon intervention with animal-based foods in the RCTs conducted in patients with MASLD.

Dietary Intervention		Liver Function Outcomes After Intervention with Animal-Based Foods											
		Hepatic Enzymes									Hepatic Steatosis		Liver Fibrosis
		AST			ALT			ALP			Hepatic Inflammation	Grade of Fatty Liver	
		B	PI	*p*	B	PI	*p*	B	PI	*p*			
[21]	**Ghee**	30.8 ± 15.8	26.7 ± 11	*p* = 0.007	42.1 ± 22.9	37.9 ± 19.3	*p* = 0.075	171.9 ± 61.7	161.7 ± 58.1	*p* = 0.029	N/A	B—Normal Liver: 0 (0%); Grade 1: 0 (0%); Grade 2: 46 (86.6%); Grade 3: 9 (13.4%); PI—Normal Liver: 0 (0%); Grade 1: 10 (18%); Grade 2: 39 (70.9%); Grade 3: 6 (10.9%);	N/A
[22]	**Bio Yogurt**	34.33 ± 13.94	38.6 ± 16.88	*p* < 0.05	51 ± 29.59	53.2 ± 33.33	NS	95 ± 30.27	98.15 ± 40.32	NS	N/A	N/A	N/A
[22]	**Conventional Yogurt**	31.94 ± 11.6	40.5 ± 23.93	NS	43.43 ± 19.98	62.75 ± 51.47	NS	110.06 ± 47.85	126.5 ± 72.33	NS	N/A	N/A	N/A
[23]	**Probiotic Yogurt Fortified with Vitamin D**	N/A	N/A	N/A	N/A	N/A	N/A	N/A	N/A	N/A	N/A	N/A	N/A
[23]	**Unfortified Probiotic Yogurt**	N/A	N/A	N/A	N/A	N/A	N/A	N/A	N/A	N/A	N/A	N/A	N/A
[24]	**Freshwater Fish**	27.29 ± 11.41	N/A	N/A	44.00 (27.50, 51.00)	21.00 (16.00, 35.00)	*p* = 0.000	N/A	N/A	N/A	N/A	Liver fat content (MRI-PDFF, %): B—18.76 ± 6.70; I—13.87 ± 7.60, *p* = 0.000	N/A
[24]	**Freshwater Fish and Red Meat**	24.82 ± 8.83	N/A	N/A	30.00 (25.50, 37.00)	28.00 (18.50, 35.00)	*p* = 0.075	N/A	N/A	N/A	N/A	Liver fat content (MRI-PDFF, %): B—17.49 ± 4.58; I—15.66 ± 5.59, *p* = 0.068	N/A
[25]	**Omega-3 Oil**	N/A	N/A	N/A	N/A	N/A	N/A	N/A	N/A	N/A	FLI: B—72.3 ± 16.0; I—68.7 ± 18.1, *p* = 0.930	N/A	N/A
[26]		N/A	N/A	N/A	N/A	N/A	N/A	N/A	N/A	N/A	N/A	N/A	N/A
[27]		0.69 ± 0.3	0.66 ± 0.2	NS	0.98 ± 0.5	0.94 ± 0.4	NS	N/A	N/A	N/A	N/A	Liver fat %: B—13.44 ± 7.7; I—12.32 ± 8.9; P—NS	FIB-4 score: B—1.36 ± 0.7; I—1.37 ± 0.7; P—NS

Data is presented as Mean ± SD, or Median (25th–75th). N/A states that there was no data available. AST, ALT, and ALP are expressed in IU/L. *p* value > 0.05 results considered as NS. AST, Aspartate aminotransferase; ALT, Alanine aminotransferase; ALP, Alkaline phosphatase; FLI, Fatty Liver Index; FIB-4, Fibrosis-4 score; MRI-PDFF, Magnetic Resonance Imaging—Proton Density Fat Fraction; SD, Standard Deviation; NS, Not significant; N/A, Not Available; B, Before Intervention; I, After Intervention; Baseline; PI, Post-Intervention.

**Table 13:** Comparisons of changes in liver function outcomes after interventions with animal-based foods and control/placebo in the RCTs conducted in patients with MASLD.

Change in the Liver Function Outcomes Between Interventions with Animal-Based Foods in MASLD							
Reference	Intervention	Hepatic Enzymes			Hepatic Steatosis		Liver Fibrosis
		AST	ALT	ALP	Hepatic Inflammation	Grade of Fatty liver	
[21]	**Ghee**	*p* = 0.119	*p* = 0.051	*p* = 0.004	N/A	*p* < 0.001	N/A
[22]	**Bio Yogurt**	*p* > 0.05	*p* > 0.05	*p* > 0.05	N/A	N/A	N/A
[22]	**Conventional Yogurt**	*p* > 0.05	*p* > 0.05	*p* > 0.05	N/A	N/A	N/A
[23]	**Probiotic Yogurt Fortified with Vitamin D**	N/A	N/A	N/A	N/A	N/A	N/A
[23]	**Unfortified Probiotic Yogurt**	N/A	N/A	N/A	N/A	N/A	N/A
[24]	**Freshwater Fish**	N/A	*p* = 0.002	N/A	MRI-PDFF, %, *p* = 0.032	N/A	N/A
[24]	**Freshwater Fish and Red Meat**	N/A	*p* = 0.002	N/A	MRI-PDFF, %, *p* = 0.032	N/A	N/A
[25]	**Omega-3 Oil**	N/A	N/A	N/A	*p* = 0.004	N/A	N/A
[26]		N/A	N/A	N/A	N/A	N/A	N/A
[27]		*p* > 0.05	*p* > 0.05	N/A	N/A	*p* > 0.05	*p* > 0.05

For each comparison, we report the *p* value and the direction of change in PI. Cell background color indicates statistical significance—green for significant results (e.g., *p* < 0.05) and red for non-significant results (*p* ≥ 0.05). Independently, the color of the numeric value (value text) reflects effect direction: green denotes a positive (favorable) change in PI, whereas red denotes a negative (unfavorable) change. N/A states that there was no data available. *p* value > 0.05 results considered as NS. AST, Aspartate aminotransferase; ALT, Alanine aminotransferase; ALP, Alkaline phosphatase; MRI-PDFF, Magnetic Resonance Imaging—Proton Density Fat Fraction; N/A, Not Available.

## Data Availability

No new data were created or analyzed in this study. Data sharing is not applicable to this article.
